# Early clinical experience with a total body irradiation technique using field-in-field beams and on-line image guidance

**DOI:** 10.1016/j.phro.2020.09.004

**Published:** 2020-10-01

**Authors:** Ruud G.H. van Leeuwen, Drean Verwegen, Peter G.M. van Kollenburg, Marc Swinkels, Richard W.M. van der Maazen

**Affiliations:** Department of Radiotherapy, Radboudumc, Huispost 874, P.O. Box 9101, 6500 HB Nijmegen, The Netherlands

## Abstract

**Background and purpose:**

Total body irradiation (TBI) is a treatment used in the conditioning of patients prior to hematopoietic stem cell transplantation. We developed an extended-distance TBI technique using a conventional linac with multi-leaf collimator to deliver a homogeneous dose, and spare critical organs.

**Materials and methods:**

Patients were treated either in lateral recumbent or in supine position depending on the dose level. A conventional linac was used with the patient midline at 350 cm from the beam source. A series of beams was prepared manually using a 3D treatment planning system (TPS) aiming to improve dose homogeneity, spare the organs at risk and facilitate accurate patient positioning. An optimized dose calculation model for extended-distance treatments was developed using phantom measurements. During treatment, in-vivo dosimetry was performed using electronic dosimeters, and accurate positioning was verified using a mobile megavoltage imager. We analyzed dose volume histogram parameters for 19 patients, and in-vivo measurements for 46 delivered treatment fractions.

**Results:**

Optimization of the dose calculation model for TBI improved dose calculation by 2.1% at the beam axis, and 17% at the field edge. Treatment planning dose objectives and constraints were met for 16 of 19 patients. Results of in-vivo dosimetry were within the set limitations (±10%) with mean deviations of 3.7% posterior of the lungs and 0.6% for the abdomen.

**Conclusions:**

We developed a TBI treatment technique using a conventional linac and TPS that can reliably be used in the conditioning regimen of patients prior to stem cell transplantation.

## 1 Introduction

In the treatment of hematological malignancies, a hematopoietic stem cell transplantation (HSCT), either autologous or allogenic, can be part of the treatment, depending on the risk profile of the disease, and age and comorbidities of the patient. Often, total body irradiation (TBI) is used in the conditioning regimen prior to HSCT [1], [2].

In TBI, a dose is given to the whole body of 2–14.4 Gy. In reduced-intensity, or non-myeloablative, schemes, a dose is given of 2–4 Gy in one or two fractions. In myeloablative schemes, a higher total dose of up to 14.4 Gy is given in up to eleven fractions [3]. A large survey from 2013 showed that for myeloablative schemes, a total dose of 12 Gy, given in six fractions (12 Gy/6fr), is used most often [4].

For the myeloablative schemes, radiation pneumonitis is a potentially lethal side effect for which occurrence can be reduced by lowering the dose given to the lungs [2], [5], [6], [7], [8], [9]. Some clinics also reduce the dose to other risk organs (kidneys, brain, eye lenses) depending on the total dose and fraction size [10], [11], [12], [13], [14].

Conventionally, TBI is performed with the patient at an extended distance (e.g., 400 cm) from the beam source. If required, sparing of organs at risk is performed, e.g., by placing lead or Cerrobend blocks where dose reduction is prescribed. Often, dose calculation is performed using hand calculations, with patient dimensions that are acquired manually [2], [15].

To improve dose homogeneity, and the accuracy of dose calculation, centers have recently started using CT scans and 3D treatment planning systems (TPS) to prepare TBI treatment plans [16], [17], [18], [19]. Instead of using cast lead blocks, the multi-leaf collimator (MLC) in the linac can be used to improve homogeneity, and to reduce dose to risk organs. Irradiation can be performed at extended source-surface distance (SSD) [20], [21], or at the standard linac isocenter using, e.g., volumetric modulated arc therapy (VMAT) techniques [2], [19], [22], [23], [24], [25].

We developed an extended-SSD field-in-field technique for TBI using a conventional linac and a 3D TPS. Patient position was verified using a commercially available mobile megavoltage imager, and accurate dose delivery was assessed by in-vivo dosimetry with electronic dosimeters. To our knowledge, this is the first reported TBI technique combining all these aspects. We aim to demonstrate our technique, and show that accurate delivery of a homogeneous dose distribution is possible using a standard linac and commercially available technology.

## 2 Materials and Methods

### 2.1 Patient selection and treatment position

We analyzed treatments of 19 patients, treated with this field-in-field technique between October 2018 and February 2020. Patients were positioned with the midline 350 cm from the source of an Elekta linear accelerator (Agility MLC, Elekta, Stockholm, Sweden), with energy 10 MV, gantry angle 273.4 degrees and collimator angle 45 degrees, enabling a maximum treatment length of 160 cm. Patients were treated with knees flexed to fit the treatment range from head to toes. Patients receiving myeloablative TBI (12 Gy/6fr, 9 Gy/2fr, 9.9 Gy/3fr) were treated in lateral recumbent position (see Supplementary Information). This position enabled separate sparing of the left and right lung without compromising the dose in the spine, sternum and mediastinal structures. Patients receiving non-myeloablative low-dose TBI (2 Gy/1fr, 4 Gy/2fr) were treated in supine position since no relative lung-sparing was needed, thereby enabling faster positioning and treatment planning.

Patients were treated on a dedicated modified treatment table using a vacuum cushion (BlueBAG, Elekta, Stockholm, The Netherlands) for positioning and reproducibility of the exact treatment position throughout simulation and treatment. A spoiler screen (perspex, thickness 1 cm) was positioned in the beam close to the patient as a buildup medium, to increase skin dose. Treatment was given at a dose rate of 0.46 Gy/min at depth 10 cm, SSD 340 cm. For all patients, position verification and in-vivo dosimetry was performed.

### 2.2 Dosimetry and quality assurance measurements

Treatment plans were prepared using the Pinnacle TPS (v16.0.2, Philips, Eindhoven, The Netherlands) at 4 mm dose resolution. For accuracy of dose calculation at long treatment distances, an optimized beam calculation model was developed, based on the standard 10MV linac model, with parameters optimized based on output and profile measurements in the treatment configuration.

These measurements were performed using a Farmer-type ionization chamber in a small closed dosimetry water phantom (15 × 23 × 23 cm), at depths of either 5 cm or 10 cm and an SSD of 340 cm (see Supplementary Information). Measurements were performed without spoiler screen. A 40 × 40 cm 10 MV beam was used at a collimator angle of 45 degrees and a gantry angle of 270 degrees. To measure diagonal profiles the phantom was moved perpendicular to the beam axis with the ionization chamber at distances from the beam axis increasing from 0 to 88 cm. To measure the dose near MLC leaf edges, the detector was positioned on the beam axis at SSD 340 cm and 10 cm depth, and a single full leaf bank was positioned at different positions near the beam axis, either blocking the measurement point or leaving it irradiated.

All measurements were simulated in the TPS to determine the necessary adjustments to the default beam model. Absolute dose values were optimized by changing the Gy/MU calibration value of the beam model, the calculated beam profile was modified by changing the flattening filter attenuation, and the penumbra and out-of-field dose of MLC fields were improved by changing the source parameters and MLC transmission value.

In-vivo dosimetry was performed using metal–oxidesemiconductor field-effect transistor (MOSFET) dosimeters (TN-502RD, Best Medical, Ottawa, Canada). These dosimeters were used to measure skin dose, however, comparison with the dose calculated by the TPS was performed using a reconstructed central dose [16]. To convert the in-vivo measured entry or exit surface dose to the central dose, we determined conversion factors dependent on the local patient radiological distance. A second-order polynomial fit was used to describe the conversion factors as a function of (radiological) distance.

The conversion factors were determined using a solid water phantom with varying thickness (6, 10, 14, 20 and 30 cm) with a Farmer-type ionization chamber at the axis of a 40 × 40 cm beam in the center of the phantom at source-detector distance (SDD) 350 cm (see Supplementary Information). The MOSFET dosimeters were positioned on the surface of the phantom at the entry and exit side, at a position near the beam axis. During the measurements, the spoiler screen was used, and small (1.5 × 1.5 × 1 cm) slabs of silicon rubber covered the dosimeters. These slabs were used during patient measurements to attenuate low-energy scattered radiation from the spoiler screen that was of limited clinical relevance but could substantially contribute to the surface dose in blocked parts of the beam.

### 2.3 CT simulation

For treatment planning, a CT scan was made using a Philips Brilliance CT Big Bore scanner (Philips, Best, NL), with the patient in lateral recumbent or supine position. The scan protocol used a 5 mm slice thickness. Before scanning, the treatment reference point was determined, ideally placed at the midrange of the body, both in the anterior-posterior, left–right, and cranio-caudal direction, and consecutively marked using three radio-opaque markers. If placing the markers at the treatment reference point was impractical, e.g., in the pubic area, a separate setup reference point was marked and offsets were documented (see Supplementary Information).

Three extra radio-opaque markers were placed on the skin to mark points for in-vivo dosimetry in the following areas, for patients treated in lateral recumbent position: posterior of the left lung (“Lung P”), lower back (“Abdomen”), and on the chest anterior of point Lung P (“Lung A”). On patients treated in supine position, three markers were placed: on the right upper arm at the level of the lungs (“Lung R”), the right hip (“Abdomen”), and on the left upper arm opposite to point Lung R (“Lung L”). After scanning, measurement points were marked using pin point tattoo marks. Further markings were applied using ink pens on both the skin of the patient and the vacuum cushion to enable accurate positioning later, throughout treatment.

### 2.4 Treatment planning

For dose prescription purposes, a Planning Target Volume (PTV) was defined in the TPS as the volume encompassed by the skin excluding the outer 5 mm. Although the skin was target of treatment, for evaluation of the dose distribution, the skin was deducted due to the uncertainties of dose calculation near the patient surface. For myeloablative treatments, the lungs were also excluded from the PTV. Dose was prescribed to the mean dose of the PTV.

For the myeloablative dose prescriptions, a reduced mean dose objective was used for the lungs to clinical tolerance levels. Depending on the dose prescription, a lowered maximum dose constraint was used for the brain and the kidneys compared to the rest of the PTV. Table 1 shows examples of dose constraints for two treatment schemes.

**Table 1:** Dose constraints for 12 Gy/6fr TBI, and for 2 Gy/1fr treatment planning prescriptions. V_11Gy_ denotes the relative volume of the organ receiving 11 Gy or more. Percentages are relative to the PTV prescription dose. For definitions of the PTV, see text.

*12 Gy/6 fr*	Mean dose aim	Min. dose	Max. dose	*2 Gy/1fr*	Mean dose aim	Min. dose	Max. dose
Lungs	10 Gy	9 Gy	V_11Gy_ < 10%	**Brain**	2 Gy	1.8 Gy (90%)	2.4 Gy (120%)
Kidneys	12 Gy	9.6 Gy (80%)	13.2 Gy (110%)	**PTV (excluding brain)**	2 Gy	1.8 Gy (90%)	2.6 Gy (130%)
Brain	12 Gy	9.6 Gy (80%)	13.2 Gy (110%)				
PTV (excluding kidney/brain)	12 Gy	9.6 Gy (80%)	14.4 Gy (120%)				

Using the optimized TBI beam model, a treatment plan was prepared in the TPS that consisted of several different beams from both the anterior (A) and posterior (P) direction (for lateral recumbent position) or left (L) and right (R) direction (for supine position). Beams were defined such that the treatment reference point was at 250 cm lateral to the linac isocenter. Furthermore, the treatment reference point was lowered by 15 cm, for positioning convenience and due to the limited maximum height of the treatment couch. The gantry angle was 273.4 degrees such that the beam axis crossed the treatment reference point. This resulted in a distance from source to treatment reference point of 350.4 cm. In the dose calculation, the spoiler screen was incorporated on all CT slices using a volume with a density override of 1.17 g/cm^3^.

**Fig. 1:**
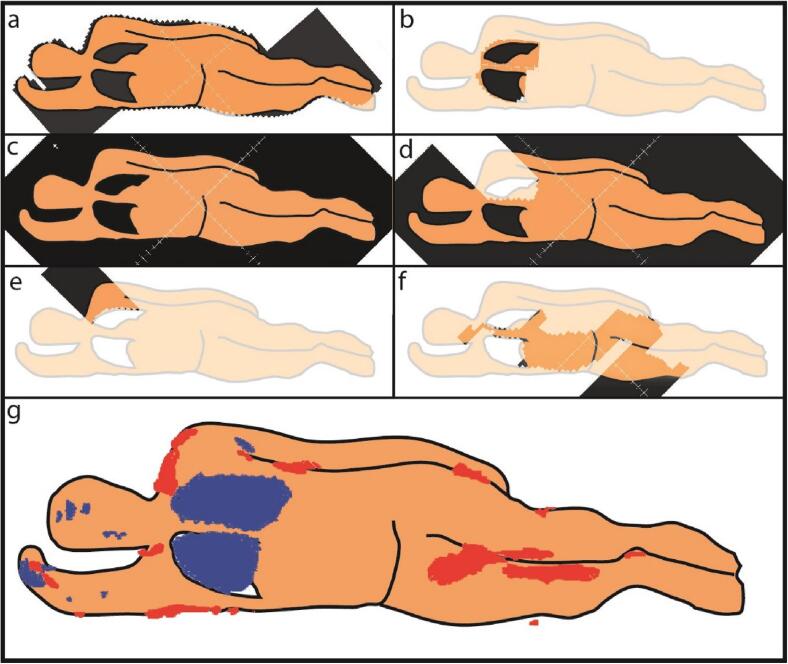
Selection of MLC fields for a 12 Gy/6 fr. TBI treatment. All shown fields were irradiated from the anterior. From top to bottom, left to right: (a) a contour field used for positioning using the light field; (b) a positioning field, used for accurate positioning using a mobile megavoltage imager; (c) an open field; (d) a field blocking the left lung (blocking of right lung not shown); (e) and (f) various fields for homogenizing the dose. Field shapes are projected on a drawing representing the patient in lateral recumbent position with knees flexed. Fields used from the posterior were similar in shape and monitor units. (g) Projection of high and low dose volumes for the TBI treatment. Red and blue patches show the projection of volumes higher than 115%/13.8 Gy, and of volumes lower than 90%/10.8 gy not including the skin (5 mm), respectively.

All treatment beams were created manually in the TPS using digitally reconstructed radiographs (DRRs) to position MLC leaves with respect to patient anatomy. Fig. 1 shows an example of a series of beams for a myeloablative TBI treatment. Treatment plans included a contour field used for positioning the patient using the light field; a positioning field slightly larger than the projection of the lungs to check the patient position prior to treatment using a mobile megavoltage imager (see Section 2.5); an open field; and fields blocking the left and/or right lung. After a first dose calculation and evaluation of the dose distribution, various additional beam segments were created in a trial and error process to homogenize the dose distribution. Using inverse optimization for this last step was not possible in the TPS due to the extended distance.

For the MOSFET measurements, the midline doses and the radiological diameter in the beam direction (AP or LR) were determined in the TPS for all three measurement positions.

We analyzed the dose distributions of 19 patients, with dose prescriptions as follows: 2 Gy/1fr: 5; 4 Gy/2fr: 3; 12 Gy/6fr: 5; 9 Gy/2fr: 5; 9.9 Gy/3fr: 1. For two patients (9 Gy/2fr and 12 Gy/6fr), lung dose was further constrained with respect to the protocol due to prior irradiation. We analyzed the dose value histogram (DVH) D95 and D5 values (dose delivered to 95%/5%) of the PTV; for the myeloablative patients we also analyzed the lung D10 value (dose delivered to 10%) and the mean lung dose. Since patients were treated with different doses, DVH values were either normalized with the prescribed PTV dose or the protocolized lung mean dose aim.

### 2.5 Patient positioning and in-vivo dosimetry

Before treatment, the patient was positioned on the treatment table using the skin markings and the room lasers, which included the default linac lasers, extra horizontal setup lasers positioned 15 cm lower than the default horizontal laser, and dedicated sagittal TBI lasers placed 250 cm lateral to the standard linac isocenter.

**Fig. 2:**
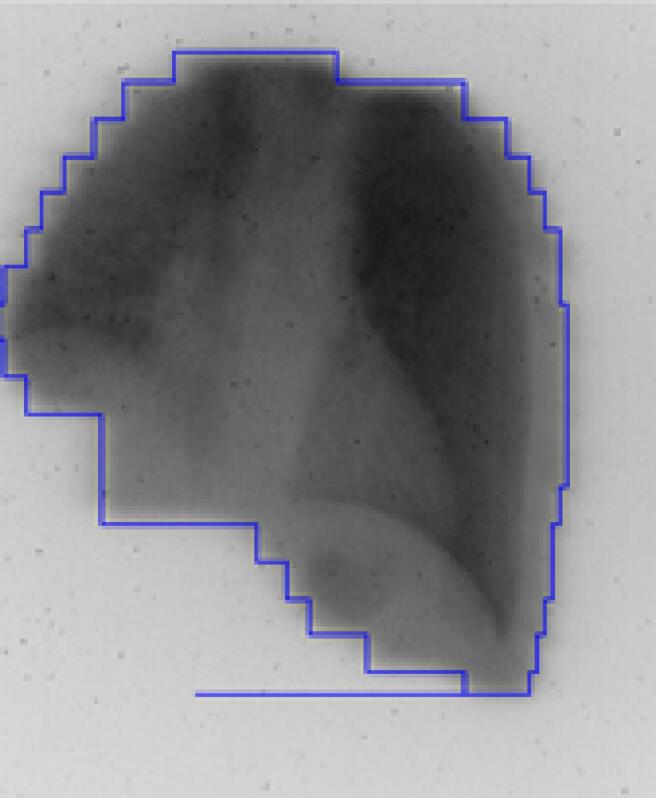
Image created using the mobile megavoltage imager and irradiation with a field similar to Fig. 1b, used for verification of the positioning of the lungs with respect to the MLC.

The position of the patient was checked using the light field of the contour field (Fig. 1a). Next, to verify the positioning of the lungs, a positioning field was irradiated and visualized using a mobile megavoltage imager (Theraview TBI, Cablon, Leusden, The Netherlands) that was positioned behind the patient. The generated image (Fig. 2) was qualitatively compared with a DRR from the TPS. If necessary, the position of the patient was corrected and verified using a second positioning field. After position verification, the treatment from that side was given. After the completion of all fields for the first side the table was turned 180 degrees and the procedure was repeated for the other side.

During treatment, MOSFET dosimeters were positioned at the marked positions, and covered with slabs of silicon rubber (1.5 × 1.5 × 1 cm) as described in Section 2.2. MOSFET measurements were recorded after the irradiation of each side, resulting in six measurements per fraction (three positions; entry and exit). Measured entry and exit doses were converted to a central dose and summed for each position. The measured doses were compared to the doses predicted by the TPS with a tolerance level of 10%. In-vivo measurements were conducted for all 19 patients however for the analysis, measurements of two patients (9 Gy/2fr and 12 Gy/6fr) were excluded due to issues with the positioning of the dosimeters for these patients. Measurements of the lung positions performed without rubber slabs were also excluded, resulting in measurements from 46 fractions.

## 3 Results

### 3.1 Optimization of TPS beam model

**Fig. 3:**
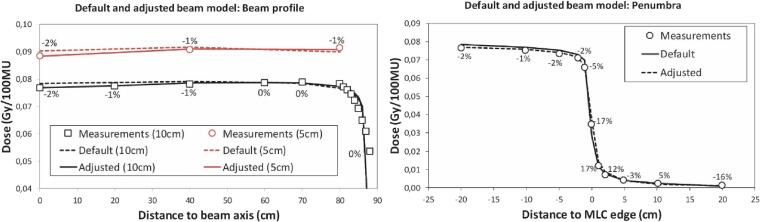
Comparison of the default Pinnacle Agility beam model with our beam model optimized for a long SSD. Data were obtained at an SSD of 340 cm. Data labels show the improvement between default and adjusted model. The distance on the horizontal axis is defined at a distance from the beam source of 350 cm. Left: Beam profile; 40 × 40 cm field measured along the diagonal. Right: Penumbra measurement, obtained by leaving the ionization chamber at the beam axis, and moving the MLC bank.

For the default beam model, the maximum deviation with the phantom measurements was 2.2% in the central 160 cm along the diagonal of a 40 × 40 cm field (SSD 340 cm, Fig. 3). At the central axis at 10 cm depth, the deviation was 2.1%. Optimizing the beam model reduced the maximum deviation to 1.4% for the diagonal profile. The deviation at the beam axis at depth 10 cm was below 0.1%.

Compared to the measurements near the MLC leaf bank (penumbra), the maximum deviations for the default model in the unblocked part and at the field edge were 5.8% and 23%, respectively. For the optimized model, the deviations were 0.9% and 5.7%. In the blocked part at 20 cm from the field edge, the deviation improved from 28% to 12% by optimizing the beam model.

### 3.2 Treated patients and dose distributions

For the non-myeloablative treatment, PTV D95 and D5 values were reached within the 80%–120% range (Table 2; note that a maximum dose of 130% was allowed for non-myeloablative treatments). For the myeloablative treatments, the D5 values remained below 120%. For all but three patients a D95 value was reached higher than the dose constraint of 80%. Two patients had tighter dose constraints for the lungs, lowering the dose in the PTV tissue directly adjacent to the lungs. For the lungs, a mean dose was reached less than 10% from the prescribed lung dose for all but the two patients with tighter dose constraints. For all patients, the Lung D10 met the dose constraint (e.g., 11 Gy for the example in Table 1).

**Table 2:** DVH parameters for non-myeloablative and myeloablative treatment plans. Dose values were normalized with the prescribed PTV dose or lung mean dose aim.

	Non-myeloablative		Myeloablative			
	**PTV**		**PTV**		**Lung**	
	**D95**	**D5**	**D95**	**D5**	**D10**	**Dmean**
N	8	8	11	11	11	11
Median	0,95	1,10	0,83	1,08	1,04	1,00
Minimum	0,90	1,10	0,44	1,06	0,58	0,51
Maximum	1,00	1,15	0,87	1,13	1,11	1,06
25% percentile	0,94	1,10	0,77	1,07	1,02	0,92
75% percentile	0,95	1,10	0,86	1,10	1,09	1,02

### 3.3 In-vivo dosimetry

**Fig. 4:**
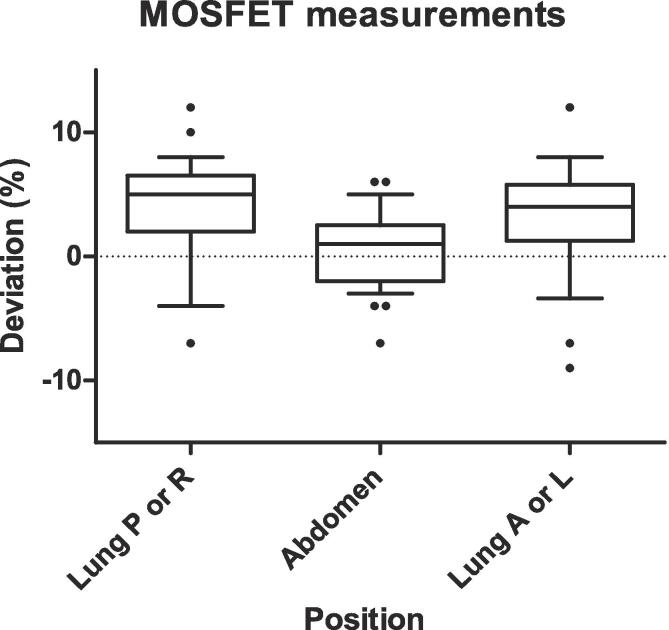
Results of MOSFET measurements. The deviation shows the difference between the reconstructed central dose for each measurement position, and the Pinnacle dose at the corresponding point. The box shows the median and 25th and 75th percentile; whiskers show the 10th and 90th percentile.

For the “Abdomen” measurement points, agreement between in and vivo measurements and dose calculation (Fig. 4) for all measurements was within the action limit interval with a mean dose deviation of 0.6%.

For the lung points, one measurement for the Lung P point and one measurement for the Lung A point exceeded the action limit of +10%. For both patients involved, values for the other fractions remained within the tolerance interval. The mean dose deviation was slightly offset (+3.7% for the Lung P/R point and +3.2% for the A/L point).

## 4 Discussion

We developed an extended-distance field-in-field TBI technique using a conventional linac. To improve homogeneity, and spare critical organs, we used the MLC of the linac. Treatment plans were prepared using a 3D TPS. Patient positioning and dose delivery were verified during treatment using a mobile megavoltage imager and MOSFET dosimeters. We analyzed 19 treatment plans, and *in vivo* measurements for 46 fractions. The DVH objectives were met for 16 patients; *In vivo* measurements were within the action limit interval for all three measurement positions for 44 fractions.

A recent publication demonstrated the experience of two centers using a similar extended-SSD treatment technique, one of them using a mobile imager, and the other using MOSFET dosimetry [21]. In our technique, we combine both these aspects, thereby more accurately monitoring dose delivery.

Compared to recently introduced VMAT techniques [2], [22], [23], [25], extended-SSD techniques are less sensitive to positioning and field junctions, since a single treatment isocenter is used, rather than three or more. Further potential issues with VMAT techniques are circulating blood volumes, and a higher dose rate. A high dose rate [8], among other factors such as total dose, fractionation, and lung shielding, has been shown to correlate with a higher risk of interstitial pneumonitis. A shortcoming of our study is that no followup data was incorporated to directly compare clinical outcomes (e.g., disease-free survival or incidence of pneumonitis) to other techniques.

For dose calculation of TBI in our TPS, the default beam model already performed reasonably well, however, as was shown previously [17], commissioning a dedicated TBI beam model improved the agreement of the dose calculation with phantom measurements.

MOSFET dosimetry showed that *in vivo*, dose calculation at the level of the abdomen was quite accurate, using the interpolation method described above. For the level of the lungs, however, the measured dose showed a mean deviation of 3.2%/3.7%, although only 2 of 46 measurements exceeded the 10% action limit. Previous reports using MOSFET dosimetry [16], [21] did not show this offset. Our measurements do not show whether this deviation is due to the simple, radiological distance-based, inhomogeneity correction that we use to convert the measured surface dose to the midline dose or, alternatively, due to the TPS inhomogeneity correction. Performance of the TPS in the lung area could be further evaluated, e.g., using measurements in a (anthropomorphic) phantom with inhomogeneities.

We used a mobile imager to position the patient, based on a projection of the thorax since that is the area most critical for toxicities. For areas outside the field-of-view of the panel, no image guidance is performed. Surface guided solutions using a 3D camera could further improve positioning of the whole body, thereby potentially enhancing accurate dose delivery. In a robustness analysis, the effect of various positioning uncertainties on the 3D dose distribution could be further assessed [25].

In conclusion, we presented our experience with an extended-SSD Field-in-field TBI technique using a conventional linear accelerator, a 3D treatment planning system, and in-vivo dosimetry and image guidance during treatment. We showed that implementation and accurate delivery is possible using commercially-available equipment.

## Declaration of Competing Interest

The authors declare that they have no known competing financial interests or personal relationships that could have appeared to influence the work reported in this paper.
