# Supplementary material for: Early clinical experience with a total body irradiation technique using field-in-field beams and on-line image guidance
Source: Phys Imaging Radiat Oncol. 2020 Oct 1;16:12–7. doi: 10.1016/j.phro.2020.09.004 (PMC7807619; doi:10.1016/j.phro.2020.09.004)
Supplement: Supplementary Data 1 [file mmc1.pdf]

Supplementary info Van Leeuwen et al. 2020

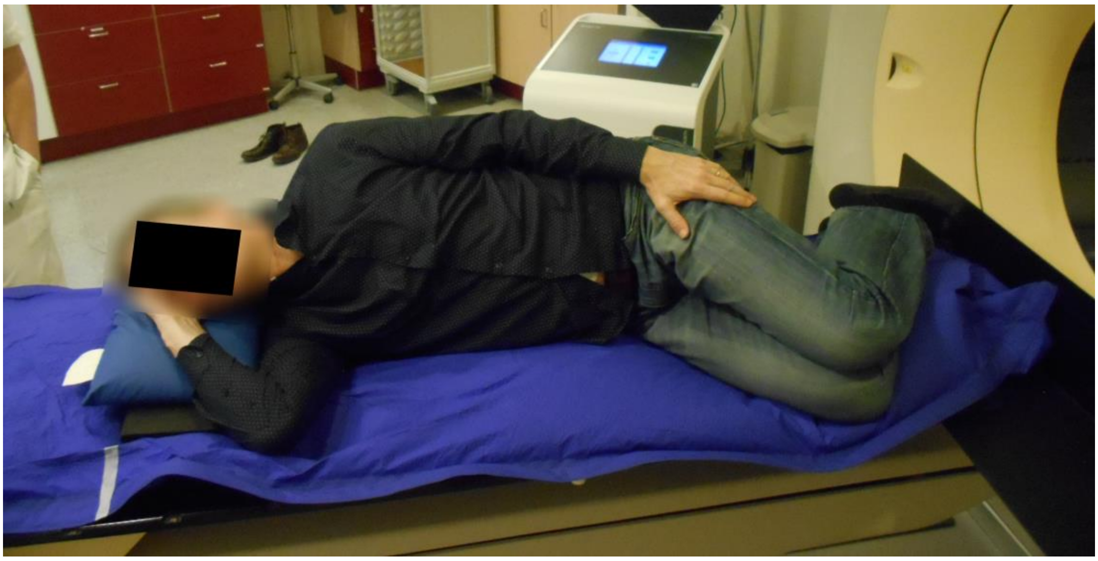

Figure 1. Volunteer in lateral recumbent position used for myeloablative TBI. Note that patients were scanned and treated in undergarment with markings on the bare skin.

o

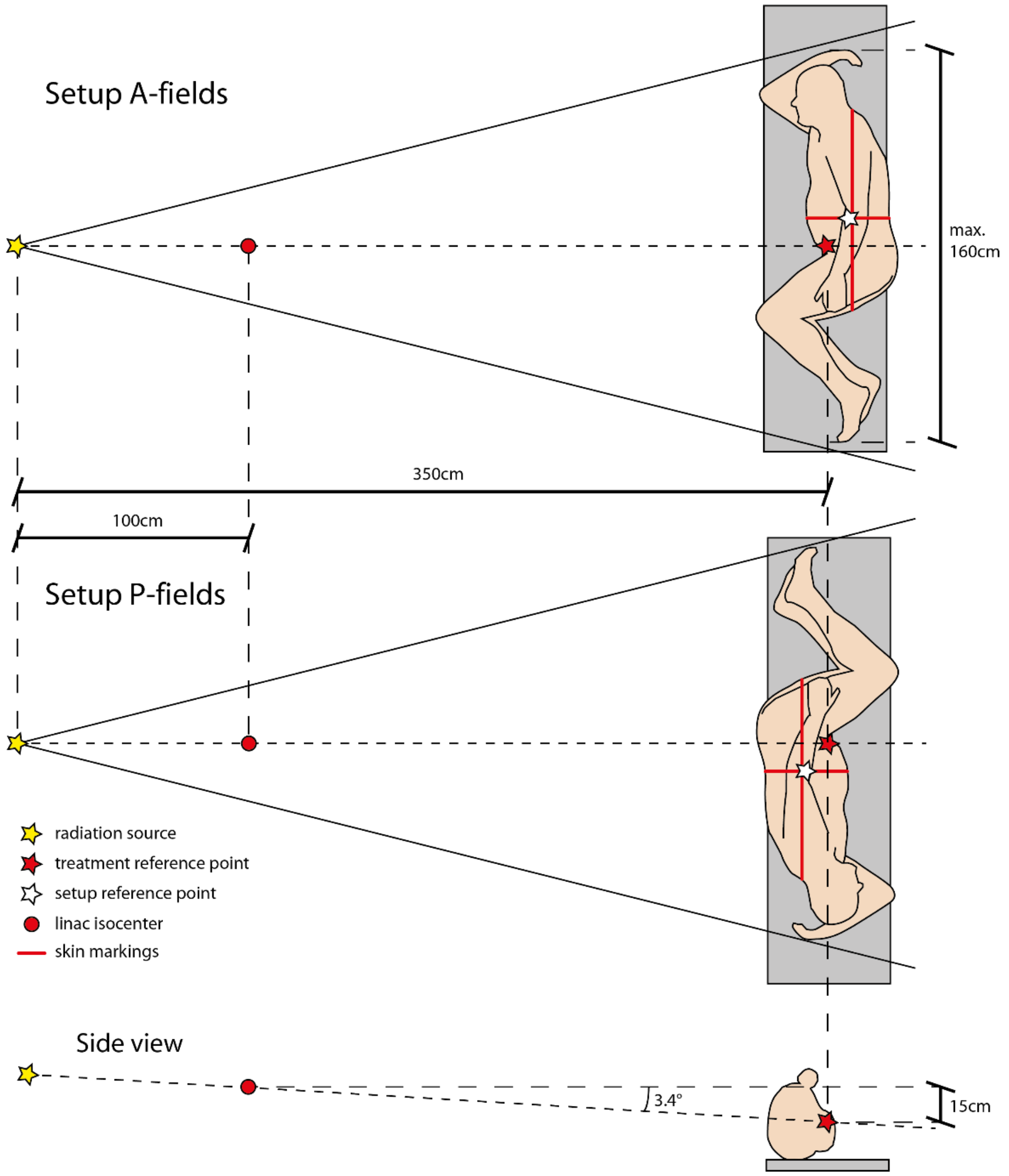

Figure 2. Setup for AP/PA TBI treatment:

1. Patient is positioned on table.

2. Patient and table are positioned using setup reference point, skin markings, and fixed room lasers, of which one at the level of the treatment reference point, 250cm from the linac isocenter. Note: setup reference point and treatment reference point are both in the same plane, 15cm lower than the linac isocenter.

3. Patient and table are moved from setup reference point to treatment reference point using documented offsets

4. AP-beams are irradiated.

5. Tabel is rotated 180 degrees and patient and table are moved to treatment reference point.

6. PA-beams are irradiated.

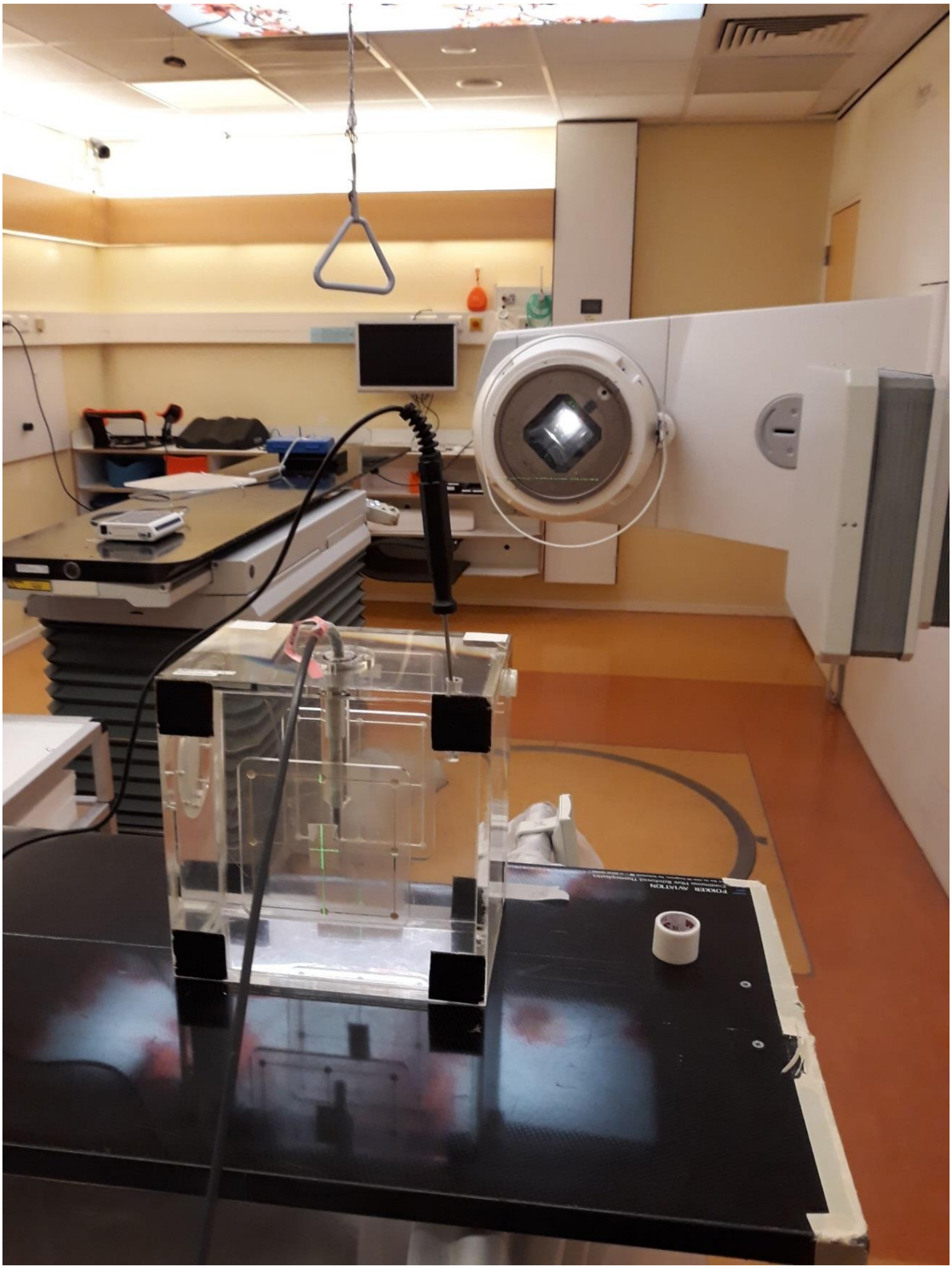

Figure 3. Ionisation chamber measurements for optimization of TPS TBI beam model (SSD 340cm, depth 10cm).

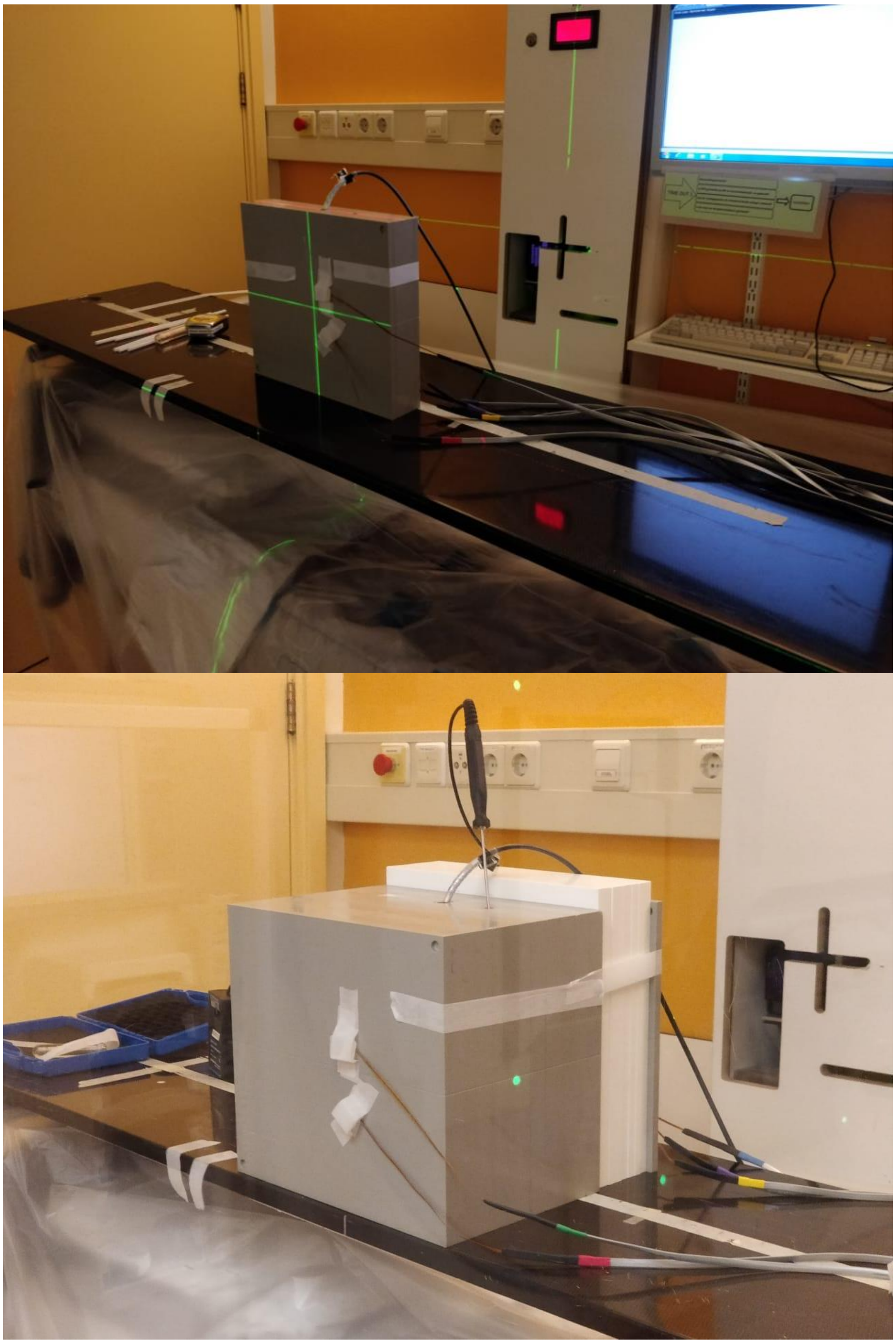

Figure 4. Measurements to determine conversion factors for in-vivo dosimetry. Top: Phantom of 6cm thickness. Bottom, phantom of 30cm thickness. In both cases, an ionization chamber in the center of the phantom is positioned on the beam axis, at source-detector distance 350 cm. MOSFET dosimeters are positioned at the entry and exit side of the phantom. Multiple dosimeters were positioned at the entry and exit side simultaneously to improve accuracy.
